# An 18-Month-Old Child with Infantile Pompe Disease: Oral Signs

**DOI:** 10.1155/2017/5685941

**Published:** 2017-03-28

**Authors:** Derya Ceyhan, Burcu Gucyetmez Topal

**Affiliations:** Department of Pediatric Dentistry, Suleyman Demirel University, Isparta, Turkey

## Abstract

We aim to create an information platform by contributing orodental findings of Pompe disease to literature. An 18-month-old male patient with Pompe disease was referred to our clinic due to swelling of the gums. In first dental examination, a nonfluctuant, normal gingiva colored swelling at the right anterior region of maxilla was detected. His parents were recommended to perform finger massage to the region. Six months later, 51, 52, 62, and 74 numbered teeth had erupted, there was a fusion between 51 and 52 numbered teeth, 84 numbered tooth was seen to be erupted, and a swelling at the site of this tooth, similar to previous one, was present. Finger massage was recommended for this area as well, and the swelling was found to have decreased at the follow-up, one week later. Tooth eruption problems and developmental dental abnormalities should be included in the signs for Pompe disease.

## 1. Introduction

Pompe disease is an unique glycogen storage disease with a lysosomal metabolism defect and autosomal recessive trait. It is due to a biochemical defect in the lysosomal acid alpha-1,4-glucosidase enzyme, encoded by glucosidase acid alpha gene on the 17. chromosome [1, 2]. This is a relatively rare, fatal disease with a reported incidence of 1 : 14.000–1 : 300.000 [1, 3]. Only one of a limited number of reports published on Pompe disease has been on dentistry [4]; among the several case reports in Turkey [5, 6], no study has yet been encountered concerning the dental findings of Pompe disease.

Pompe disease has infantile, juvenile, and adult forms, and the most severe form is the fatal infantile form [7]. The signs of the disease are progressive muscle weakness, hypotonia, myopathy, respiratory failure, hepatosplenomegaly, difficulty in feeding, aphonia, weakness in tongue, macroglossia, and inability to control oral secretions [8].

This report presents the case of an 18-month-old patient who was diagnosed as Pompe disease. In the limited number of accessible reports on Pompe disease, orodental findings have rarely been encountered. In this case report, we aim to present orodental signs of Pompe disease and contribute to the literature.

## 2. Case Report

An 18-month-old male patient with Pompe disease was referred to our clinic due to swelling of his gingiva while he was being treated by enzyme replacement therapy in the intensive care unit of the university hospital. During the anamnesis, it was learned that the patient had a birth weight of 3.270 g from a normal term pregnancy and cesarean section, and his parents were second-degree relatives. The other child of the family had died at 16 months due to Pompe disease, and the patient had a 16-year-old healthy brother.

The medical history of the patient revealed severe muscle hypotonia, respiratory problems, gastric reflux, and a mild cardiomyopathy. His respiration was through a ventilator and he was fed by gastrostomy tube; he was conscious and cooperative but could not speak.

As a result of genetic assessment of the peripheral blood sample at the genetic department of another university, it was learned that a homozygous c.1408_1410 del mutation had been detected.

**Figure 1:**
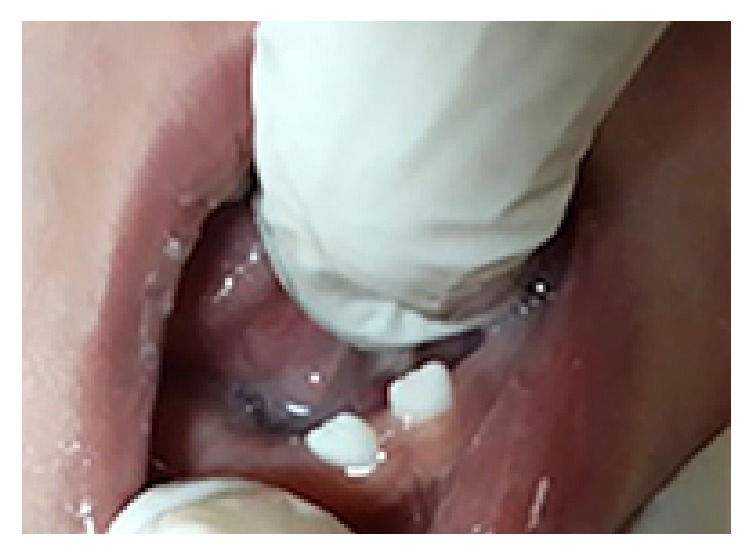
The patient's mandibular anterior segment and erupted 71 and 81 numbered teeth.

**Figure 2:**
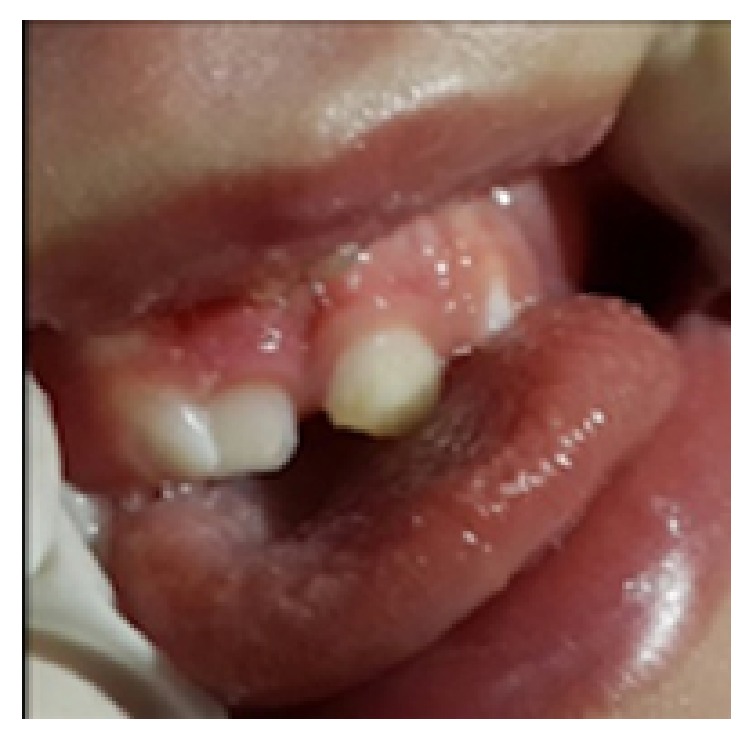
At the examination six months later, erupted 51, 52, and 62 numbered teeth, and the fusion between 51 and 52 numbered teeth, and the enamel fracture at the mesial edge of 61 numbered tooth.

**Figure 3:**
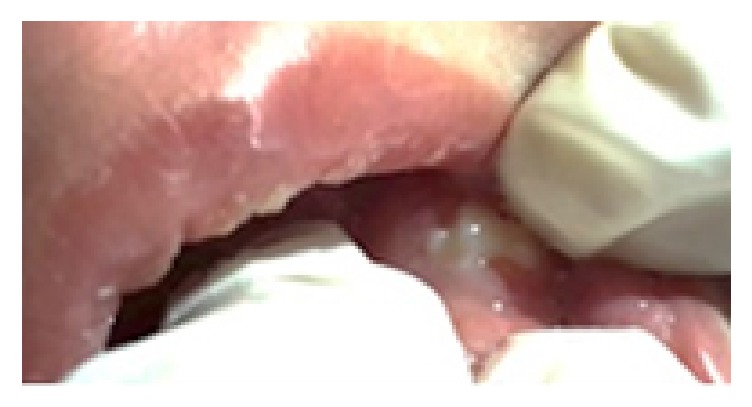
Newly erupted 74 numbered tooth.

His first dental examination was performed by us when he was hospitalized at the intensive care unit. His mouth was found to be open due to macroglossia and respiratory failure, and a nonfluctuant, normal gingiva colored swelling at the right anterior region of the maxilla was detected. No color change, focus of bleeding, cystic formation, or infection was encountered at the region. Tooth eruption was noted for 61, 71, and 81 numbered teeth (Figure 1). His anamnesis, obtained from his mother, revealed that gingival swelling was also present when 61 numbered tooth erupted. His parents were recommended to perform finger massage to the region of 51 numbered tooth. At the examination six months later, 51, 52, 62, and 74 numbered teeth had erupted (Figures 2 and 3), and there was fusion between 51 and 52 numbered teeth, and an enamel fracture at the mesial edge of 61 numbered tooth, which his mother was unaware of it, was thought to be secondary to intubation, and 84 numbered tooth was seen to be erupted and it was learned that a swelling at the site of this tooth, similar to the previous one, was present. His parents were recommended to finger massage to this area as well, and no antibiotics or anti-inflammatory drugs were prescribed during this process. The swelling was found to have decreased at the follow-up, one week later.

Symptoms of the patient disappeared overall; on the other hand, parents of the patient could not bring the patient to us after leaving the intensive care unit. Therefore, medical doctors of the patient were informed about the necessity of our follow-ups when the patient was brought to the hospital once again. The follow-up process will provide clarifying the uncertain oral signs.

## 3. Discussion

Pompe disease has an autosomal recessive trait, and prenatal diagnosis is generally possible [8]. In this case, although it was a consanguineous marriage and they had lost another child due to the same disease, the parents had undergone no prenatal tests or genetic evaluation. It is important for individuals with a consanguineous marriage, who have Pompe disease in their family history, to be in contact with physicians and to have the necessary tests conducted.

The primary teeth which should have erupted in a 24-month-old child could not be seen in the mouth of the case, and it could not be detected whether they were congenitally deficient or delayed, since no radiography was taken. These patients can be examined with the suspicion of tooth agenesis, and a radiography and/or mutational analysis of the genes frequently handled in tooth agenesis could be added to the evaluation for a definitive diagnosis. De Gijt et al. [4] reported delayed tooth eruption in the primary molar teeth of a 3-year-old patient who was brought with repeated gum swellings and diagnosed as Pompe disease; however, they reported that the eruption of the permanent teeth of the patient was normal during the follow-up. Their histopathological examinations suggested that glycogen accumulation in the fibroblasts might have caused gingival swelling; however, since a significant difference was seen in the glycogen accumulation only in the arterial smooth muscle cells, the etiology of the gingival swelling could not be completely clarified. Chronic inflammation, dry mouth, and glycogen accumulation in the fibroblasts have all been considered to play a role in the etiology of swelling [4]. Fusion between 51 and 52 numbered teeth was influent in the eruption problem that our patient had in anterior segment. However, the other gingival swelling and tooth eruption problems of our patient could be considered to be due to glycogen accumulation in the fibroblasts.

No report has been encountered in the literature on developmental tooth abnormalities in patients with Pompe disease, but Baccetti et al. [9] reported the presence of taurodontism of the primary dentition and craniofacial disproportion in the patients with glycogen storage disease type III and explained this by the anomalies in the gene content of the X chromosome or less specific abnormalities in chromatinic material development. So, the tooth eruption problems, fusion and probable hypodontia, and developmental dental abnormalities that we observed in the case should be included in the signs for Pompe disease. Furthermore, rapidly progressive periodontal disease, oral ulcerations, delayed tooth eruption, and gingival inflammation had been reported for glycogen storage disease type I [9].

In a study, only a small proportion of the children had caries experience, but most were found to have plaque on both primary and permanent teeth, associated with the inadequate oral hygiene and eating uncooked cornstarch [10]. Our patient has not any caries or plaque, related to feeding with gastrostomy tube.

We consider that the orodental signs of Pompe disease are inadequately emphasized in the limited number of accessible sources in the literature. The fact that Pompe disease is not one of the subjects which dentists commonly come across may be due to the fact that patients with Pompe disease are hospitalized in the intensive care units, their life expectancy is short, and the disease is very rarely seen. We hope that, by presenting information on the oral signs of Pompe disease with this case report, a source can be provided in order to assist in the diagnosis and treatment of children with this disease and to establish dentistry care for those patients.
